# 10-Hydroxy-2-decenoic Acid, the Major Lipid Component of Royal Jelly, Extends the Lifespan of *Caenorhabditis elegans* through Dietary Restriction and Target of Rapamycin Signaling

**DOI:** 10.1155/2015/425261

**Published:** 2015-02-19

**Authors:** Yoko Honda, Yoko Araki, Taketoshi Hata, Kenji Ichihara, Masafumi Ito, Masashi Tanaka, Shuji Honda

**Affiliations:** ^1^Department of Genomics for Longevity and Health, Tokyo Metropolitan Institute of Gerontology, Sakaecho, Itabashiku, Tokyo 173-0015, Japan; ^2^Nagaragawa Research Center, Api Company Limited, Nagara, Gifu 502-0071, Japan; ^3^Research Team for Mechanism of Aging, Tokyo Metropolitan Institute of Gerontology, Sakaecho, Itabashiku, Tokyo 173-0015, Japan

## Abstract

Royal jelly (RJ) produced by honeybees has been reported to possess diverse health-beneficial properties and has been implicated to have a function in longevity across diverse species as well as honeybees. 10-Hydroxy-2-decenoic acid (10-HDA), the major lipid component of RJ produced by honeybees, was previously shown to increase the lifespan of* Caenorhabditis elegans.* The objective of this study is to elucidate signaling pathways that are involved in the lifespan extension by 10-HDA. 10-HDA further extended the lifespan of the* daf-2* mutants, which exhibit long lifespan through reducing insulin-like signaling (ILS), indicating that 10-HDA extended lifespan independently of ILS. On the other hand, 10-HDA did not extend the lifespan of the* eat-2* mutants, which show long lifespan through dietary restriction caused by a food-intake defect. This finding indicates that 10-HDA extends lifespan through dietary restriction signaling. We further found that 10-HDA did not extend the lifespan of the long-lived mutants in* daf-15*, which encodes Raptor, a target of rapamycin (TOR) components, indicating that 10-HDA shared some longevity control mechanisms with TOR signaling. Additionally, 10-HDA was found to confer tolerance against thermal and oxidative stress. 10-HDA increases longevity not through ILS but through dietary restriction and TOR signaling in* C. elegans*.

## 1. Introduction

One of the most important challenges in the study of aging is the discovery of compounds with longevity-promoting activities and the elucidation of their underlying mechanisms. Such compounds could provide potential nutraceutical or pharmaceutical approaches to slow aging and the onset of age-related diseases in humans [1]. Royal jelly (RJ) is produced by the hypopharyngeal, postcerebral, and mandibular glands of the worker bees and has been implicated to be involved in the longer lifespan of queens in contrast with workers in the honeybee* Apis mellifera* L., because queens are fed throughout their lives with RJ, whereas workers are fed RJ for only a short period of time during their larval stage [2–5]. RJ has been also reported to extend the lifespan of nematodes [6, 7], flies [8, 9], and mice [10] indicating that RJ has a common role in longevity across phyla. RJ comprises proteins, sugars, lipids, vitamins, and free amino acids, together with a variety of bioactive substances [11]. The identities of the components that play critical roles in longevity are not fully understood. A single protein, termed royalactin contained in RJ, was reported to extend the lifespan of* D. melanogaster* and* C. elegans* [9, 12]. We previously found 10-hydroxy-2-decenoic acid (10-HDA), which is the major lipid component of RJ [13] and has several health-beneficial effects in mammals [14], such as antitumor activity [15], anti-inflammatory activity [16], and antiangiogenic activity [17]. It also extends the lifespan of* C. elegans* [6]. However, how 10-HDA extends lifespan is not well elucidated.

It has been reported that lifespan is regulated mainly through insulin-like signaling (ILS) and dietary restriction signaling in* C. elegans* as well as* Drosophila melanogaster* and other experimental animals [18]. In the present study, we found that 10-HDA extended the lifespan of* C. elegans* not through ILS but through dietary restriction signaling. Dietary restriction signaling has been reported to mediate lifespan extension through various downstream signaling pathways including target of rapamycin (TOR) signaling [19]. We suggest in this report that 10-HDA extends the lifespan via the TOR signaling.

## 2. Methods

### 2.1. Culture and Strains of* C. elegans*

*C. elegans* strains were maintained at 20°C on nematode growth medium agar with* Escherichia coli* OP50 as a food source, as previously described [20]. The N2 Bristol strain was used as the wild type* C. elegans*. The mutant strains used in this study were CB1370:* daf-2(e1370)* III; DA465:* eat-2(ad465)* II; CB138:* unc-24(e138)* IV; DR412:* unc-24(e138)/daf-15(m81)* IV; and LG344:* geIs8[gpa-4p::skn-1b::gfp *+* rol-6(su1006)]*.

### 2.2. Treatment with 10-HAD

10-HDA was purchased from Alfresa Pharma Co., Ltd., Osaka, Japan. 10-HDA was added to liquid NGM that had been autoclaved and cooled to 50°C, using DMSO as a solvent at a final concentration of 0.03%. The media were immediately dispensed into Petri dishes. 10-HDA was provided at 20°C from adult 0-day to death in the lifespan assay and from hatched L1 stage in the progeny production assay. Experiments involving 10-HDA were conducted in parallel with those involving a control group treated with an equivalent volume of DMSO.

### 2.3. Lifespan Determination

Lifespan was determined as previously described [6]. UV-killed* E. coli* strain OP50 was used as a food source in the experiment to avoid any effects of live* E. coli* on 10-HDA and any effects of 10-HDA on growth and metabolism of live* E. coli*. Lifespan under UV-killed* E. coli* is longer compared to that under live* E. coli* probably because of the diminished toxicity of growing bacteria [21]. Worms were raised until the L4 molt and were then transferred onto a new plate containing 40 *μ*M 5-fluoro-2′-deoxyuridine (Sigma Aldrich, St. Louis, MO, USA) to prevent self-fertilization [22, 23]. The day of transfer at the L4 molt was counted as adult 0-day. Worms were judged to be dead when they did not respond to a mechanical stimulus. To focus on aging, worms that had become desiccated on the side of the plate after crawling off or those that displayed extruded internal organs were excluded from our analysis. The results of the survival assays were analyzed using the Kaplan-Meier method, and significance was measured with the log-rank test using the statistical analysis package StatMate III (ATMS, Tokyo, Japan).

### 2.4. Measurement of Progeny Production

Single newly hatched N2 worm was placed on a plate containing UV-killed OP50. Worms were transferred every day or every other day to fresh plates. The resulting progenies were left to develop for 2 days for measurement of progeny number.

### 2.5. Assays of Stress Resistance

To assess thermotolerance, young adult hermaphrodites were placed on NGM plates at 35°C and scored for viability. To assess oxidative stress, young adult hermaphrodites were placed on NGM plates, which included 50 mM paraquat (Sigma Aldrich) at 20°C and were scored for viability.

## 3. Results

### 3.1. 10-HDA Extends Lifespan Independently of ILS

**Figure 1:**
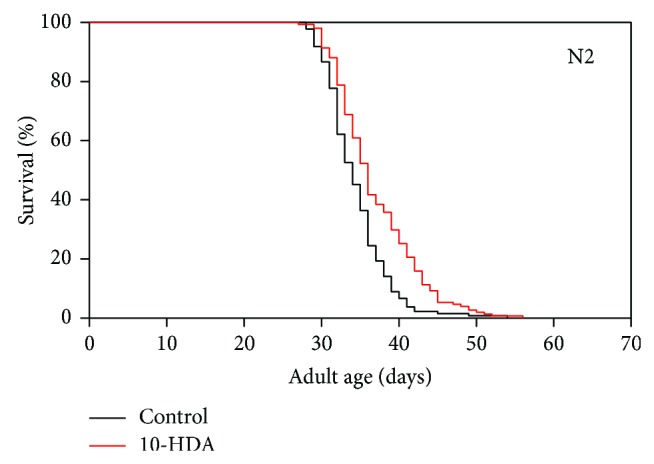
The effect of 10-HDA on the lifespan of N2 wild type* C. elegans*. The curves shown in Figures 1, 2, 3, and 5 represent pooled data from three to four experiments (Table 1).

**Figure 2:**
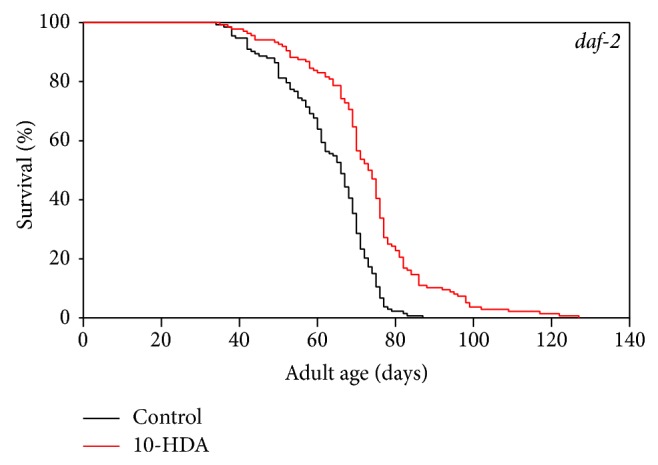
The effect of 10-HDA on the lifespan of the* daf-2* mutants.

As shown in Figure 1 and Table 1, 10-HDA extended the lifespan of N2, a wild type strain of* C. elegans* as previously described [6]. To investigate whether this lifespan extension effect of 10-HDA was due to ILS, we evaluated the effect of 10-HDA on the lifespan of the insulin-like receptor* daf-2* mutants, which reduce ILS and show long lifespan [18]. We found that 10-HDA further extended the lifespan of* daf-2* (Figure 2 and Table 1) indicating that 10-HDA affected lifespan independently of ILS. The previous finding that 10-HDA extended the lifespan of the mutants in* daf-16,* which encodes the FOXO transcription factor, the downstream target of ILS [6], also supports this notion. Alternatively, 10-HDA appeared to extend the lifespan of the* daf-2* mutants to a greater extent than that of the wild type (Figure 2 and Table 1) suggesting some interaction between 10-HDA and ILS.

### 3.2. 10-HDA Extends Lifespan through Dietary Restriction Signaling

**Figure 3:**
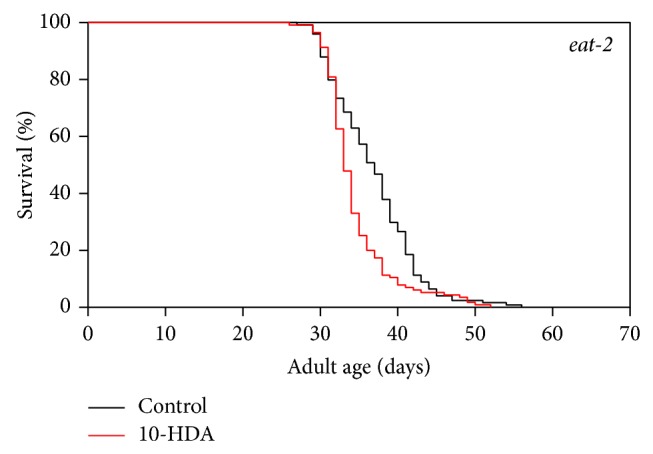
The effect of 10-HDA on the lifespan of the* eat-2* mutants.

**Figure 4:**
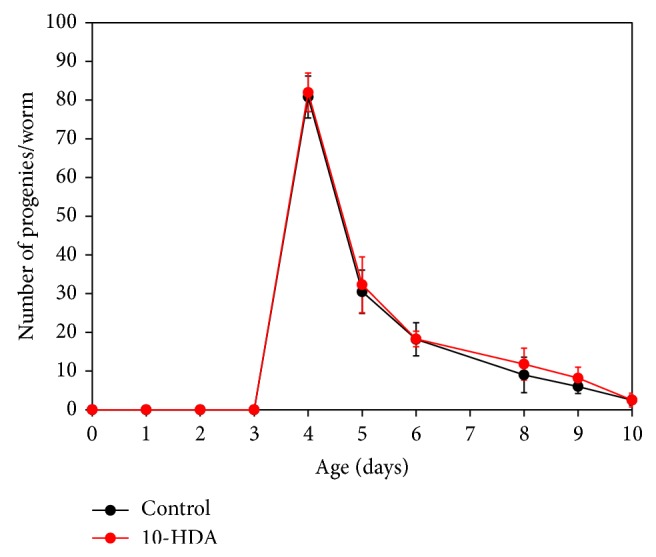
The effect of 10-HDA on the progeny production of wild type* C. elegans*. The progeny production is plotted against age at transfer. Mean values and SD are shown (*n* = 6). Day 0 corresponds to the hatched L1 stage.

To determine whether the lifespan extension by 10-HDA is mediated through dietary restriction signaling, we examined the effect of 10-HDA on the lifespan of the* eat-2* mutants, which display the extended lifespan through the feeding impairment-based dietary restriction [24]. As shown in Figure 3 and Table 1, 10-HDA did not further extend the lifespan of the* eat-2* mutants, suggesting that 10-HDA shared common lifespan control mechanisms with dietary restriction signaling. We wondered if 10-HDA directly exerts dietary restriction. Dietary-restricted worms are known to produce progeny in a delayed manner [17]. 10-HDA-treated worms produced progeny in a similar manner to untreated ones (Figure 4), suggesting that 10-HDA is unlikely to directly restrict diet. Taken together, these findings suggest that 10-HDA extended lifespan by affecting the downstream process of the dietary restriction signaling.

### 3.3. 10-HDA Extends Lifespan through TOR Signaling

**Figure 5:**
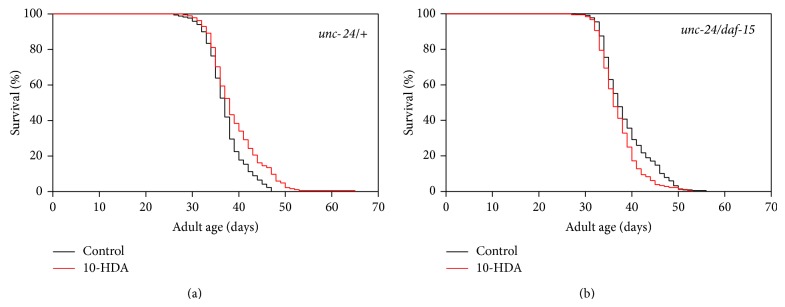
The effect of 10-HDA on the lifespan of the* daf-15* mutants. (a)* unc-24/*+ and (b)* unc-24/daf-15*.

Dietary restriction has been reported to extend lifespan through various downstream signalings including TOR signaling [15]. To determine whether 10-HDA extended lifespan via the TOR signaling, we evaluated the effect of 10-HDA on the lifespan of mutants in* daf-15*, which encodes Raptor (the regulatory associated protein of mTOR), a component of the TOR complex [25]. The* daf-15* homozygous mutants show larval arrest and heterozygotes display lifespan extension [22]. As shown in Figure 5(b) and Table 1, 10-OHDA did not further extend the lifespan of the* daf-15* heterozygous mutants; 10-HDA extended the lifespan of the control* unc-24/*+ mutants (Figure 5(a) and Table 1). This finding suggests that lifespan extension by 10-HDA is mediated through TOR signaling.

### 3.4. 10-HDA Confers Tolerance against Thermal and Oxidative Stresses

**Figure 6:**
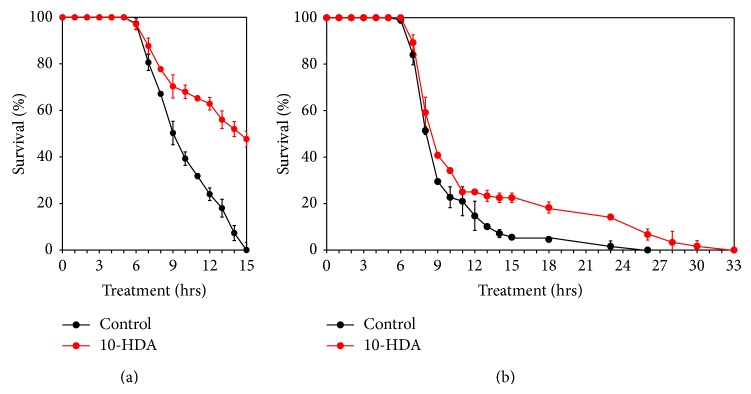
(a) Thermal stress. (b) Oxidative stress. Treatment with or without 25 *μ*M 10-HDA began at the egg stage. Mean values and SD of triplicate samples are plotted.

To assess whether 10-HDA confers tolerance against thermal and oxidative stresses, we examined the effects of 10-HDA treatment on survival during heat exposure and paraquat exposure. As shown in Figures 6(a) and 6(b), 10-HDA increased survival during both heat exposure and paraquat exposure.

## 4. Discussion

The findings we have presented here suggest that 10-HDA, the major lipid component of RJ, extends the lifespan of* C. elegans* through dietary restriction and TOR signaling. RJ is believed to have several components with lifespan-extending activity, including peptides (6), royalactin (9, 12), and 10-HDA, which work through a variety of signaling pathways, such as ILS, EGF, or TOR. This seems to indicate that RJ originally has diverse functions in honeybees, such as cast differentiation, queen longevity, and nourishment.

A variety of compounds have been shown to extend the lifespan of* C. elegans* [1]. Among them, anticonvulsants like ethosuximide [26], valproic acid [27], icariin [28], and caffeic acid phenethyl ester [29] extend lifespan via ILS. The present study showed that 10-HDA further extended the lifespan of* daf-2* mutants that showed long lifespan via ILS, indicating that 10-HDA extends lifespan independently of ILS.

On the other hand, dietary restriction mimetics including 2-deoxyglucose extend lifespan like direct dietary restriction [30]. Further, several compounds that target downstream of dietary restriction signaling also extend lifespan: *α*-ketoglutarate via the TOR signaling [31]; diallyl trisulfide [32] via SKN-1 pathway; metformin via AMP-activated protein kinase (AMPK); and SKN-1 pathways [33]. The SKN-1 transcription factor is the ortholog of mammalian Nrf2 and known to control stress protection. 10-HDA was found to confer tolerance against thermal and oxidative stresses (Figures 6(a) and 6(b)). However, we failed to find the induction of* skn-1* by 10-HDA treatment using the reporter of* skn-1*:* geIs8[gpa-4p::skn-1b::gfp*] (unpublished observation), suggesting that 10-HDA may not be involved in the SKN-1 pathway. Metformin has also been reported to retard aging by directly affecting metabolism of folate and methionine in* E. coli*, the worm's food [34]. Resveratrol extended lifespan via Sir2 [35] and AMPK pathways [19]. However, resveratrol has also been reported not to extend the lifespan of worms or flies [36] or mice [37]. Furthermore, oxaloacetate extended worm lifespan via both IIS and dietary restriction [38] but was reported not to extend mouse lifespan [37].

In the present study, we found that 10-HDA extended* C. elegans* lifespan via dietary restriction and the TOR pathway. RJ, whose major lipid component is 10-HDA, plays an important role in an epigenetic fate determination between worker and long-lived queen honeybees with identical genome composition [2–5, 39]. TOR has been reported to participate in the caste fate determination in honeybees [40–42]. Although it is unknown whether 10-HDA is involved in the caste fate determination, it may be relevant that 10-HDA functions as an inhibitor of histone deacetylase, which is known to play a main role in epigenesis [43] and that 10-HDA affects the expression of histone deacetylase 3 and DNA methyltransferase 3, both of which also play a role in epigenesis in honeybees [44].

TOR is known to be a nutrient sensor and controls lifespan as well as protein synthesis and degradation, cell growth, and autophagy [45–48]. Rapamycin, which inhibits TOR, extends the lifespan of yeasts [49], nematodes [50], flies [51], and mice [52]. However, chronic administration of rapamycin in rodents has several side effects such as glucose intolerance, insulin tolerance, and cataracts [53–57]. It may be possible that 10-HDA contained in RJ which is widely taken as a health food could be used as nutraceutical intervention aimed at mammalian TOR (mTOR) inhibition to delay aging and the onset of age-related diseases.

## 5. Conclusions

The present study indicates that 10-HDA extends lifespan of* C. elegans* not via ILS but via dietary restriction and the TOR signaling. It may be possible that 10-HDA contained in RJ which is widely taken by human as a health food could be used as nutraceutical intervention aimed at mTOR inhibition to delay aging and the onset of age-related diseases.

## Figures and Tables

**Table 1:** The effects of 10-HDA on the lifespan of various mutants.

Exp.	Genotype	Treatment	Mean life span ± SEM (days)	% change	Maximum life span (days)	% change	*N* (animal)	Significance
1	N2	Control	34.8 ± 0.8	+11	54	−4	34	*P* < 0.01
	N2	10-HDA 25 *µ*M	38.7 ± 1.0		52		26	

2	N2	Control	34.4 ± 0.6	+10	49	+14	45	*P* < 0.01
	N2	10-HDA 25 *µ*M	37.7 ± 0.8		56		29	

3	N2	Control	34.3 ± 0.5	+6	45	+11	56	*P* < 0.01
	N2	10-HDA 25 *µ*M	36.2 ± 0.5		50		95	

1	*daf-2 *	Control	61.3 ± 12.3	+10	82	+20	31	*P* < 0.01
	*daf-2 *	10-HDA 25 *µ*M	67.2 ± 11.7		98		34	

2	*daf-2 *	Control	63.7 ± 10.5	+24	87	+46	49	*P* < 0.01
	*daf-2 *	10-HDA 25 *µ*M	79.2 ± 17.5		127		41	

3	*daf-2 *	Control	63.0 ± 11.9	+14	83	+18	53	*P* < 0.01
	*daf-2 *	10-HDA 25 *µ*M	71.8 ± 13.9		98		61	

1	*eat-2 *	Control	32.5 ± 2.8	+6	39	+25	23	
	*eat-2 *	10-HDA 25 *µ*M	34.5 ± 4.4		49		21	

2	*eat-2 *	Control	39.8 ± 4.8	−13	56	−14	30	
	*eat-2 *	10-HDA 25 *µ*M	34.8 ± 5.1		48		11	

3	*eat-2 *	Control	37.0 ± 5.3	−7	54	−4	71	
	*eat-2 *	10-HDA 25 *µ*M	34.4 ± 4.4		52		83	

1	*unc-24/+ *	Control	37.8 ± 0.8	+4	46	+9	58	
	*unc-24/+ *	10-HDA 25 *µ*M	40.2 ± 1.1		50		61	
	*unc-24/daf-15 *	Control	39.6 ± 1.3	−5	49	−14	71	
	*unc-24/daf-15 *	10-HDA 25 *µ*M	36.4 ± 0.6		42		65	

2	*unc-24/+ *	Control	36.6 ± 0.5	+4	46	+9	58	*P* < 0.01
	*unc-24/+ *	10-HDA 25 *µ*M	38.1 ± 0.6		50		61	
	*unc-24/daf-15 *	Control	37.7 ± 0.5	−5	49	−14	71	
	*unc-24/daf-15 *	10-HDA 25 *µ*M	35.8 ± 0.4		42		65	

3	*unc-24/+ *	Control	37.7 ± 0.6	+6	47	+13	43	*P* < 0.01
	*unc-24/+ *	10-HDA 25 *µ*M	39.8 ± 0.7		53		47	
	*unc-24/daf-15 *	Control	39.1 ± 0.6	−5	53	−11	85	
	*unc-24/daf-15 *	10-HDA 25 *µ*M	37.2 ± 0.4		47		83	

4	*unc-24/+ *	Control	36.5 ± 4.6	+7	46	+11	38	*P* < 0.01
	*unc-24/+ *	10-HDA 25 *µ*M	39.0 ± 5.8		51		41	
	*unc-24/daf-15 *	Control	37.8 ± 5.3	−3	50	−10	37	
	*unc-24/daf-15 *	10-HDA 25 *µ*M	36.5 ± 4.1		45		39	
